# Human immunodeficiency virus accelerates brain aging and disrupts the trajectory of glymphatic clearance in aging brain

**DOI:** 10.3389/fpsyt.2025.1509093

**Published:** 2025-05-28

**Authors:** Benedictor Alexander Nguchu, Jing Zhao, Yu Lu, Yifei Han, Han Jin, Xiaoxiao Wang, Hongjun Li, Peter Shaw

**Affiliations:** ^1^ Oujiang Laboratory (Zhejiang Lab for Regenerative Medicine, Vision and Brain Health), Wenzhou Medical University, Wenzhou, Zhejiang, China; ^2^ School of Ophthalmology and Optometry and Eye Hospital, Wenzhou Medical University, Wenzhou, Zhejiang, China; ^3^ Department of Radiology, Beijing Youan Hospital, Capital Medical University, Beijing, China; ^4^ School of Electronic Engineering, Tianjin University of Technology and Education, Tianjin, China; ^5^ Center for Biomedical Imaging, University of Science and Technology of China, Hefei, Anhui, China

**Keywords:** HIV-infection, chronic inflammation, accelerated brain aging, brain predicted age difference, glymphatic clearance trajectory, DTI-ALPS index, motoric and executive dysfunction

## Abstract

**Introduction:**

Existing evidence indicates that HIV enters the nervous system in the early days of infection. However, the involvement of HIV in the pathogenesis of key biological aspects of the brain, such as glymphatic clearance and brain aging, and its role in explaining complex phenomena like motoric and executive dysfunction, remains unrecognized.

**Methods:**

Herein, we recruited 145 subjects to study the brain aging using brain-predicted age differences (brain-PADs) and investigate how HIV affects the typical trajectory of glymphatic clearance in aging brain. The assessment of glymphatic clearance in the aging brain was performed using a technique called "diffusion tensor image analysis along the perivascular space” (DTI-ALPS). We further evaluated the association between accelerated brain aging trajectories and cognitive performance to explain impairments observed in motor and executive functions in people living with HIV.

**Results:**

Our results showed that subjects with HIV had increased brain-PAD in several brain structures compared to those who were HIV-negative, suggesting underlying neuropathology associated with HIV. The brain structures demonstrating accelerated aging (increased brain-PAD) include the middle frontal gyrus, pre-and post-central gyri, supramarginal gyrus, precuneus, cuneus, parietal lobule and operculum, and superior and middle occipital gyri of the left hemisphere. While normal subjects maintained typical trajectories of glymphatic clearance (as measured by the DTI-ALPS index) with age or brain-PADs for several structures, including the left central operculum, left frontal operculum, left opercular inferior frontal gyrus, and left triangular inferior frontal gyrus, none of these trajectories were maintained in subjects with HIV. Our data also show that increased brain-PAD in brain regions was associated with lower performance in motor and executive functions.

**Discussion:**

These findings suggest that HIV infection accelerates brain aging and disrupts the trajectory of glymphatic clearance in aging brain, which may explain the complex mechanisms underlying cognitive impairment in motor and executive domains often seen in HIV patients. These new insights may shift our understanding of HIV pathology and aid the development of new therapeutic targets, contrary to previous approaches.

## 1 Introduction

HIV infection has been reported to enter the brain parenchyma in the early days of the infection. Valcour et al. (1) demonstrate that evidence of HIV entry into the brain can be detected as early as 8 days after infection (1). Whether HIV entry into the brain disrupts the glymphatic profile and its trajectory with age remains unknown and unexplored. The glymphatic system is the most recently discovered system of the brain that removes metabolic waste and is demonstrated to be more active during sleep (2–4). The system allows cerebrospinal fluid (CSF) to flow from the network of perivascular spaces (PVSs) into the brain tissues via AQP4 water channels at astrocytic end-feet. This process facilitates the exchange between CSF and interstitial fluid, enabling the flushing out of toxins such as beta-amyloid and tau proteins (5), which are implicated in neurodegenerative diseases like Alzheimer’s disease(AD).

In this study, we seek to determine whether the standard trajectory of glymphatic clearance in the aging brain is disrupted by HIV infection and whether this disruption plays a role in accelerating brain aging and exacerbating cognitive dysfunction in subjects with HIV. So far, we know that the glymphatic clearance function exhibits a well-defined trajectory as we age. Typically, the glymphatic clearance function declines gradually with age. The study by Taoka et al. (6) provides evidence for this phenomena using MRI-based analyses. Using the technique called “diffusion tensor image analysis along the perivascular space (DTI-ALPS),” the authors quantified the trajectory of glymphatic clearance function in aging subjects, -and showed that normal individuals at a young age exhibit greater performance in glymphatic function (evidenced by greater DTI-ALPS) and that this function decreases significantly with advanced age (evidenced by decreased DTI-ALPS). Findings from the studies by Dai et al. (7)Wang et al. (8) Zhang et al. (9), and Matsushita et al. (10) also align with these early findings by Taoka et al. (6). They found a significant reduction in DTI-ALPS values in older subjects compared to younger subjects, indicating preserved glymphatic performance at a young age, which declines significantly with age (7, 8, 10, 11). Therefore, whether this trajectory is preserved or altered in HIV infection remains the focus of this study. Understanding this aspect would offer new insights into how HIV pathology evolves and affects brain systems. This would also provide a shift in thinking on how to address HIV-related pathology, contrary to previous approaches.

The basis for conducting this study is the fact that the glymphatic system plays a critical role in maintaining stable homeostasis of the brain, - providing a healthy environment for glial and neural cells, as well as stable conditions for immune responses. This coordinated system of the brain prevents the accumulation and the buildup of neurotoxins. The aggregation of neurotoxins promotes neurodegeneration in diseases such as AD. In HIV, the presence of glymphatic dysfunction might have great consequence, including exacerbating cognitive impairment. It is likely to cause the disruption of homeostasis and working conditions of cells carrying out cognitive tasks. Accumulated debris/neurotoxins may suffocate cells and accelerate the loss of functional ability of the neurons, inducing impairment in the executing cognitive tasks.

One possible pathway for glymphatic impairment in HIV may involve direct viral infection of the blood-brain barrier (BBB) and astrocytes, a compromised immune response, and chronic neuroinflammation (12). The viral attacks on the BBB can impact vascular permeability and pulsatility, CSF composition, and its circulation in the CNS. Constant activation of immune response through interleukin-6 (IL-6) and tumor necrosis factor-alpha (TNF-α) to recruit immune cells-such as leukocytes- to the site of infection can also impair vascular permeability and affect vascular pulsatility and astrocytic support function. The immune response is also accompanied by increased vasodilation, which can lead to excess fluid accumulation in the brain parenchyma, penetrated alongside the immune cells during immune cell recruitment. The increased fluid accumulation may disrupt the interstitial pressure gradients and the balance of convective flow for waste transport. Attacks on astrocytes, either directly or indirectly via inflammatory mediators, can suppress AQP4 and alter its expression, localization, and function (12). These alterations can lead to a loss of astrocytic function and changes in CSF flow and dynamics. People with HIV, on the other hand, are reported to have disturbed sleep. Two factors—depression and treatment side effects—have been associated with the disruption of sleep patterns in HIV infection. It has been demonstrated that proper sleep promotes glymphatic clearance and that waste removal is critical during sleep when the parenchymal extracellular space expands. Therefore, disturbances in sleep for people with HIV can have significant consequences on the critical removal of brain waste. Considering these potential risk factors for glymphatic impairment in HIV, it is rational to critically evaluate and understand the actual glymphatic profile and its trajectory in people with HIV, as well as how it may potentially affect brain aging or exacerbate cognitive dysfunction in HIV-infected individuals.

The current research demonstrates a difference between the chronological age and biological age of the brain (13, 14). The biological age, often predicted from neuroimaging data (15), can index premature brain aging and the underlying neurobiology or pathology affecting overall brain health, including cognitive function (16). Studies indicate that a greater brain-predicted age difference (brain-PAD), quantified as the difference between brain-predicted age and chronological age, can serve as a proxy measure of accelerated brain aging (14). Given this, we expand our study to determine how the disruption of glymphatic health due to HIV may accelerate brain aging. Since recent studies have established a strong relationship between glymphatic clearance and cognitive function, with reduced glymphatic function associated with decreased cognitive performance (17, 18), we believe that insights from this evaluation will guide researchers in identifying how glymphatic clearance, brain aging process, and cognitive health are interwoven in the complex inflammatory condition of HIV infection.

Here we recruited 145 individuals, including 100 HIV-1 positive subjects and 45 healthy controls, to assess the complex interplay between glymphatic function, brain aging, and cognitive function in HIV infection. The assessment of glymphatic performance was conducted using the DTI-ALPS method, which is based on diffusion imaging of MRI data. We used deep learning models to evaluate the brain-predicted ages of brain structures. These predicted ages were used to index the true biological ages of the brain structures, as opposed to the phenotypical/chronological age. The estimation of the ages of the brain structures was based on their observed sizes with reference to the general population. The cognitive performance of our subjects was assessed using self-reports and a battery of neuropsychological tests. Six domains of cognition were evaluated, with Frascati rating scales of 2007 being used to define HAND status. In the end, we compared the reports of their assessments between those with HIV and healthy subjects and examined potential interplay and associations of these different biological aspects of the brain.

## 2 Materials and methods

### 2.1 Participants

We enrolled 145 subjects for this study at Beijing YouAn Hospital, the capital Hospital, after obtaining each participant’s written informed consent. The Ethical Committee of the Capital Medical University and the University of Science and Technology of China approved the study. The procedures and experiments of this study complied with the code of Ethics of the World Medical Association (Declaration of Helsinki) for human experiments. Before conducting experiments, individuals with any of the following records were excluded from participation: Signs of neurological disorders, brain injury, brain lesions, cerebral atrophy or illicit drugs and alcohol use. Participants who were HIV + were administered antiretroviral therapy, which include the combination of tenofovir (TDF), lamivudine (3TC), and efavirenz (EFV). We then collected the blood of each patient for blood assays to assess viral load (i.e., the number of copies of HIV per millilitre of blood (copies/ml)), CD4+ T-cell counts (i.e., the number of CD4 T cells), and CD4+/CD8+ ratios. We next administered a battery of neuropsychological tests to each patient to evaluate their cognitive profiles. See **Supplementary Table S1** for participants’ demographics and a summary of clinical reports.

### 2.2 Neuropsychological testing

The assessment of cognitive performance was based on self-reports and a battery of neuropsychological (NP) tests. We identified patients at risk of cognitive impairment by assessing six cognitive domains and used the Frascati rating scales of 2007 to define HAND (19). The first domain was attention and working memory, tested using the Paced Auditory Serial Addition Test (PASAT), Continuous Performance Test Identical Pairs (CPT-IP), and the Wechsler Memory Scale-III (WMS-III). The next domain was verbal and language, tested using the category fluency and animal naming tests, while motor function was tested using the Grooved Pegboard test. We next used the Wisconsin Card Sorting Test-64 (WCST-64) to test the performance of abstract and executive function, and the Hopkins Verbal Learning Test-Revised (HVLT-R) and the Brief Visuospatial Memory Test-Revised (BVMT-R) to test the performance of learning and recall. Lastly, we assessed the performance of information processing speed using the trail-making test part A. We next standardized the raw scores of each test and obtained demographically adjusted T-scores. For cognitive domains evaluated by multiple tests, we calculated the final composite T-score of a domain by averaging the T-scores of all tests performed on the domain. We classified a patient as having ANI if, every day, regular functioning was intact, but at least two cognitive domains showed impairment in the NP test results.

### 2.3 MRI neuroimaging

The imaging of our participants’ brains was performed on a Siemens 3T MRI Scanner (Allegra, Siemens Medical System, Erlangen, Germany). The machine was equipped with a 32-channel head coil. We administered two imaging protocols to our participants: The 3D-T1-weighted and diffusion-weighted imaging protocols. We set the following configurations for 3D-T1-weighted image: TR/TE = 1,900 ms/2.52 ms, inversion time = 900 ms, flip angle = 9°, field of view (FOV) = 250 mm^2^ × 250 mm^2^, matrix size = 246 × 256, slice thickness = 1 mm, and voxel size =1 × 1× 1 mm^3^; and the following configurations for diffusion-weighted image: 60 diffusion-encoded (*b* = 1,000 s/mm^2^), 3 references (*b* = 0 s/mm^2^), TR = 3,300 ms, TE = 90 ms, flip angle = 90°, slice thickness = 4.2 mm, voxel size = 2 × 2 × 4.2 mm^3^.

### 2.4 Pre-processing of neuroimaging data

Data preprocessing steps were in line with earlier studies (9, 20, 21) We first corrected for distortions in the imaging data, typically induced by magnetic susceptibility, eddy currents, and subject movements. We used specialized tools in the FMRIB Software Library (FSL) (https://fsl.fmrib.ox.ac.uk/) (22) for this purpose. We next fitted diffusion tensors by using DTI-tensor fitting to derive quantitative metrics of diffusion properties of water molecules within different tissues. These metrics include tensors, fractional anisotropy (FA), mean diffusivity (MD), axial diffusivity (AD), and radial diffusivity (RD). To reduce the influence of individual variations and enable the evaluation of the data at the same standard space, we registered each subject’s FA map to a standard space defined by the Illinois Institute of Technology (IIT). We specifically used version 3.0 of IIT Human Brain Atlas Template (23). For this registration, we used the FMRIB’s Linear/Non-linear Image Registration Tools (FLIRT/FNIRT) from the FSL version 5.09. The choice of the IIT v3.0 template is based on its high signal quality and contrast, as evidenced by the fractional anisotropy (FA) values (visit: https://www-p-64.iit.edu/~mri/DTItemplatecomp.html). This allows for visualization of minute white matter structures and other spatial features at a resolution of 1× 1 × 1 mm^3^, making the IIIT v3.0 template the suitable choice for registration purposes. After registration, two experienced researchers (BAN and YL) visually inspected the registered images to assess the quality of registration. Having been satisfied with the registration quality, we transformed all the remaining diffusion tensor maps into the space defined by the IIT v3.0 template using a transformation matrix derived from normalizing FA maps.

### 2.5 DTI-ALPS processing and evaluation

There is well-established evidence corroborating the relationship between measures of cognitive function and scores of glymphatic function estimated by the DTI-ALPS index (9, 24). Most studies demonstrate that this relationship is more evident for the DTI-ALPS index estimated within a PVS area defined by 5 mm-diameter ROIs. On this basis, our study focuses solely on the DTI-ALPS index evaluated within this context (five mm-diameter ROIs), to examine the contribution of HIV pathology to the well-established relationship between aging and glymphatic performance, as well as to the relationship between accelerated brain aging and glymphatic dysfunction. The detailed procedures for DTI-ALPS evaluation have been documented in earlier studies (18, 24, 25). Briefly, the DTI-ALPS index is computed from diffusion tensor image. We first delineated the ROIs on standard space defined by the JHU-ICBM DTI-81 atlas. This was done by placing a 5-mm diameter spherical ROI at the center of the reference slice in the areas of intersection between the projection and association fibers adjacent to the lateral ventricles. This is the area where the medullary veins run perpendicular to the ventricle wall on the axial plane, where the glymphatic function is estimated. The placement of the ROIs was performed in both hemispheres, guided by referencing color-coded FA maps. Two qualified neurologists, YL and BAN, independently assessed the areas of ROI placement in each image. All ROIs and labels on this atlas, encompassing the key fiber areas (i.e., the projection and association fibers), were then registered to the standard space defined by the IIT version 3.0 template. From these areas defined by 5-mm diameter ROIs, we estimated the DTI-ALPS index, established to represent the glymphatic clearance function (21, 24). In essence, the DTI-ALPS index represents the water diffusivity of projection and association fiber areas along the x-axis (Dxpro, Dxasc), modulated by the water diffusivity of both the projection fiber areas (along the y-axis, Dypro) and the association fiber areas (along the z-axis, Dzasc). Mathematically, this index is given as “mean (Dxpro, Dxasc)/mean (Dypro, Dzasc)”.

### 2.6 Estimation of brain-predicted age and differences

The estimation of brain-predicted ages for brain structures was in accordance with the previous studies (26, 27). We utilized a fully automated pipeline proposed by Manjón et al. (28), called BrainStructureAges (BSA), implemented in volbrain (https://www.volbrain.net/) (28). We used this pipeline on T1-weighted images to obtain estimates of biological ages of brain structures. We leveraged the deep learning models integrated in this pipeline for this purpose. At first, the AssemblyNet model (29) was used to segment the brain structures, and the age of each structure was determined. Each structure’s age was used in the process to estimate the global biological age of the subject. This information was recorded for further analysis. Each imaging data set took about 6 to 12 minutes to process. To estimate how far the predicted biological ages differ from chronological age, we computed the difference between chronological age and brain-predicted ages of each brain structure, as well as the global biological age of the subject. This difference is referred to as brain-predicted age difference or brain-PAD and serves as a proxy measure for the underlying neurological changes occurring in the brain.

### 2.7 Statistical analysis

All statistical analyses were performed on the R v4.2.0, an environment for statistical computing (30). In the analysis, we adopted several strategies to minimize the number of statistical comparisons while ensuring that statistical assumptions were not violated. The first strategy involved comparing the brain-predicted ages of those who were HIV-positive (n = 100) with those who were HIV-negative (n=45). This strategy was employed to test the impact of HIV on accelerated brain aging, as estimated through brain-predicted age. Here we used independent t-tests for the data that met the assumptions of normality and homogeneity of variance (Levene’s test); otherwise, we applied Wilcoxon Rank sum Tests (Mann Whitney U Tests). The second strategy involved conducting linear regressions. Here the trends in the relationships between DTI-ALPS glymphatic scores and subjects’ chronological ages or brain-predicted ages of each structure were evaluated. Next, the nature of these trends in the HIV group was compared against those in the HC group. This strategy was employed to test whether HIV infection disrupts the typical relationship between glymphatic performance and chronological or brain-predicted age. The third strategy involved assessing the relationships between brain-predicted ages, particularly brain-PADs, and cognitive performance or blood clinical markers of HIV progression (viral load, CD4 T-cells, and CD4/CD8 ratio). Here we used Pearson’s or Spearman correlations, depending on the nature of the data, to test the hypothesis of the relationship while controlling for sex. Furthermore, we explored whether participant demographics and health conditions could influence the outcomes, specifically examining how ART status (including suppressive effects), duration of HIV, and HAND status impact our findings. For all analyses, the false discovery rate (FDR) was applied for multiple comparison corrections. Both β coefficients and P values were two-tailed estimates, with p <.05 set as the criterion for statistical significance.

## 3 Results

### 3.1 HIV impact on accelerated brain aging (brain-PADs)

**Figure 1:**
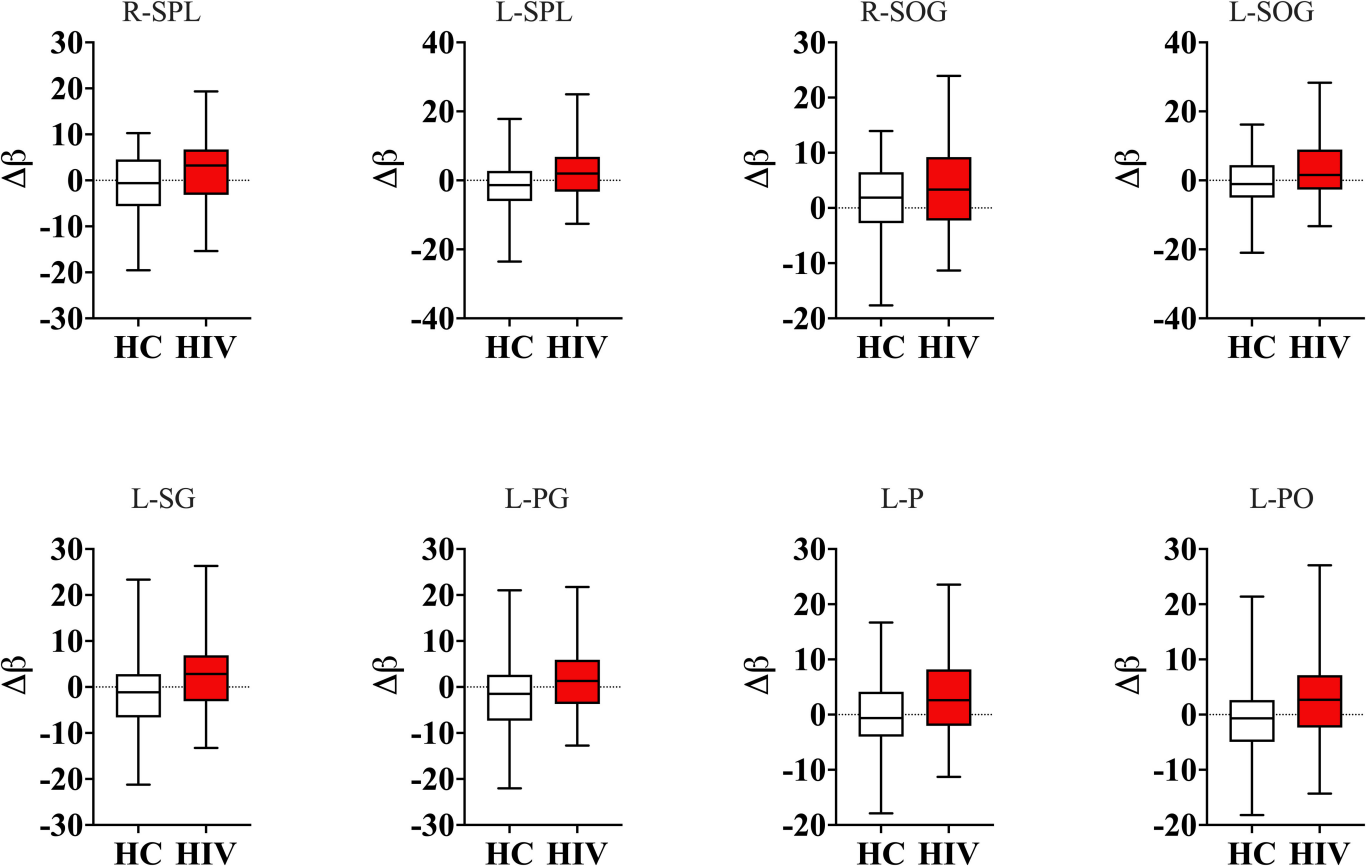
Group comparisons of Brain-Predicted Age Differences. Subjects with HIV had greater brain-predicted age differences (brain-PAD) in several brain structures compared to healthy controls. Boxplot in red shows a relative difference between predicted age of the brain structure and chronological age in subjects with HIV, representing a deviation of a biological age from the chronological age, whereas the boxplot in white shows the age differences in normal subjects. On average the normal subjects had age differences centered at zero, reflecting almost no much deviation of structural predicted age from chronological age whereas those with HIV had brain-PADs centered above zero. R-SPL, right superior parietal lobule; L-SPL, left superior parietal lobule; R-SOG, right superior occipital gyrus; L-SOG, left superior occipital gyrus; L-SG, left supramarginal gyrus; L-PG, left postcentral gyrus; L-P, left precuneus; L-PO, left parietal operculum; Δβ, relative difference between predicted age of brain structure and chronological age.

Our results showed that subjects with HIV had increased brain-PAD in several brain structures compared to those who were HIV-negative (**Figure 1**, also see **Table 1**). These findings suggest the presence of underlying neuropathology associated with HIV, which accelerates brain aging. Increased brain-PAD was more evident (P = 0.00*) bilaterally in the superior parietal lobule, superior occipital gyrus, and middle frontal gyrus, and laterally in the left supramarginal gyrus, left postcentral gyrus, left precuneus, left parietal operculum, left occipital pole, left precentral gyrus medial segment, left postcentral gyrus medial segment, left middle occipital gyrus, left cuneus, and left angular gyrus.

**Table 1:** Brain-predicted age differences (relative differences) in HC and HIV.

Brain region	HC	HIV	t	*P*-value
	Mean	Mean		
External CSF	1.1367	3.3030	-2.1391	0.0351
Right caudate	1.5456	3.8532	-2.0155	0.0468
Left caudate	0.8136	3.4104	-2.2805	0.0249
Right cerebral White Matter	1.1770	3.2417	-2.1047	0.0378
Left cerebral White Matter	-0.0564	2.8772	-2.6146	0.0104
Right lateral ventricle	1.9222	3.9904	-1.9985	0.0485
Left lateral ventricle	0.6850	3.5024	-2.6226	0.0102
Left thalamus	1.9747	4.1408	-2.0107	0.0472
Right anterior cingulate gyrus	0.7915	3.3329	-2.0872	0.0398
Left anterior cingulate gyrus	0.5902	3.1837	-2.1530	0.0341
Right angular gyrus	0.3426	2.9545	-2.4308	0.0168
Left angular gyrus	-0.3398	3.9043	-2.8904	0.0048
Left calcarine cortex	0.8616	3.5595	-2.2609	0.0258
Left central operculum	-0.7365	1.8437	-2.2342	0.0278
Left cuneus	0.1415	3.5660	-2.6514	0.0093
Left inf. occipital gyrus	0.0349	2.8518	-2.0870	0.0394
Right middle cingulate gyrus	-0.2666	2.9022	-2.7676	0.0069
Left middle cingulate gyrus	-0.6550	2.6796	-2.8593	0.0053
Right middle frontal gyrus	-0.7862	1.7704	-2.1012	0.0383
Left middle frontal gyrus	-0.8004	2.0379	-2.0536	0.0433
Left middle occipital gyrus	0.0049	4.0393	-2.6472	0.0095
Right postcentral gyrus medial segment	-0.3698	2.8861	-2.4057	0.0184
Left postcentral gyrus medial segment	-1.5460	2.2565	-2.7178	0.0080
Right precentral gyrus medial segment	-0.5620	2.3295	-2.3372	0.0219
Left precentral gyrus medial segment	-1.3044	2.1382	-2.6815	0.0089
Right sup. frontal gyrus medial segment	-0.8021	2.1818	-2.2897	0.0245
Left sup. frontal gyrus medial segment	-0.7432	2.2407	-2.2963	0.0241
Left occipital pole	-1.4673	2.6005	-2.9942	0.0034
Right opercular inf. frontal gyrus	0.0262	2.2057	-2.0612	0.0417
Right posterior cingulate gyrus	2.0429	4.1878	-1.9860	0.0500
Left posterior cingulate gyrus	1.0839	3.9260	-2.5453	0.0125
Right precuneus	1.1086	3.6794	-2.1806	0.0318
Left precuneus	-0.0432	3.3288	-2.7608	0.0069
Left parietal operculum	-0.7758	2.7623	-2.7915	0.0064
Right postcentral gyrus	-1.0661	1.8123	-2.5676	0.0117
Left postcentral gyrus	-1.9293	1.9224	-2.8431	0.0056
Right precentral gyrus	-1.0965	1.5408	-2.3348	0.0216
Left precentral gyrus	-1.8534	1.6530	-2.6123	0.0107
Left planum temporale	-0.5982	2.4693	-2.5789	0.0114
Right sup. frontal gyrus	-1.4639	1.5881	-2.3026	0.0238
Left sup. frontal gyrus	-1.6565	1.5813	-2.3847	0.0195
Right supplementary motor cortex	-1.4572	1.6927	-2.4707	0.0156
Left supplementary motor cortex	-1.8174	1.6589	-2.6397	0.0100
Right supramarginal gyrus	-0.8607	1.8786	-2.5691	0.0116
Left supramarginal gyrus	-1.0263	2.9963	-2.7923	0.0064
Right sup. occipital gyrus	1.3040	3.9876	-2.0804	0.0401
Left sup. occipital gyrus	-0.9681	3.2879	-2.9624	0.0038
Right sup. parietal lobule	-0.5396	2.8222	-2.6600	0.0092
Left sup. parietal lobule	-1.7774	2.4156	-2.9202	0.0044
Left sup. temporal gyrus	-0.0039	2.5638	-2.1991	0.0302
Left transverse temporal gyrus	-0.2918	2.2098	-2.2212	0.0286

^*^the values in HC and HIV are the means of the brain-predicted differences for each region; t and p are the statistical scores for independent two-sample t-tests.

### 3.2 The link between accelerated brain aging and glymphatic dysfunction

**Figure 2:**
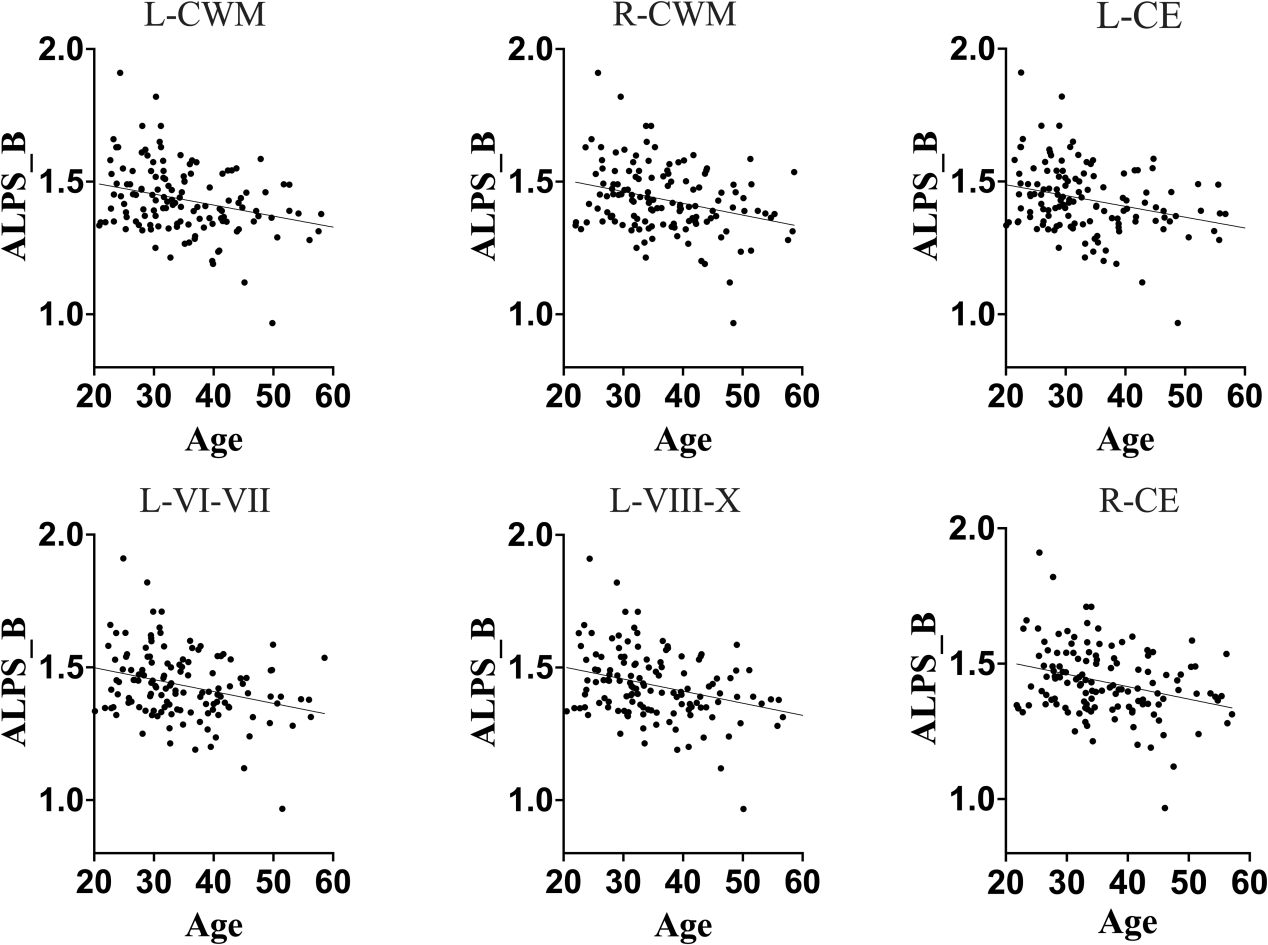
Association between DTI-ALPS index of glymphatic clearance and predicted ages of brain structures in full sample. Generally, DTI-ALPS index of a full sample was negatively correlated with predicted age of brain structures. These results suggest that, in general population, the glymphatic clearance declines as the brain ages in general population. L-CWM, left cerebellum white matter; R-CWM, right cerebellum white matter; L-CE, left cerebellum exterior; L-VI-VII, lobules VI-VII; L-VIII-X, lobules VIII-X; R-CE, right cerebellum exterior.

**Figure 3:**
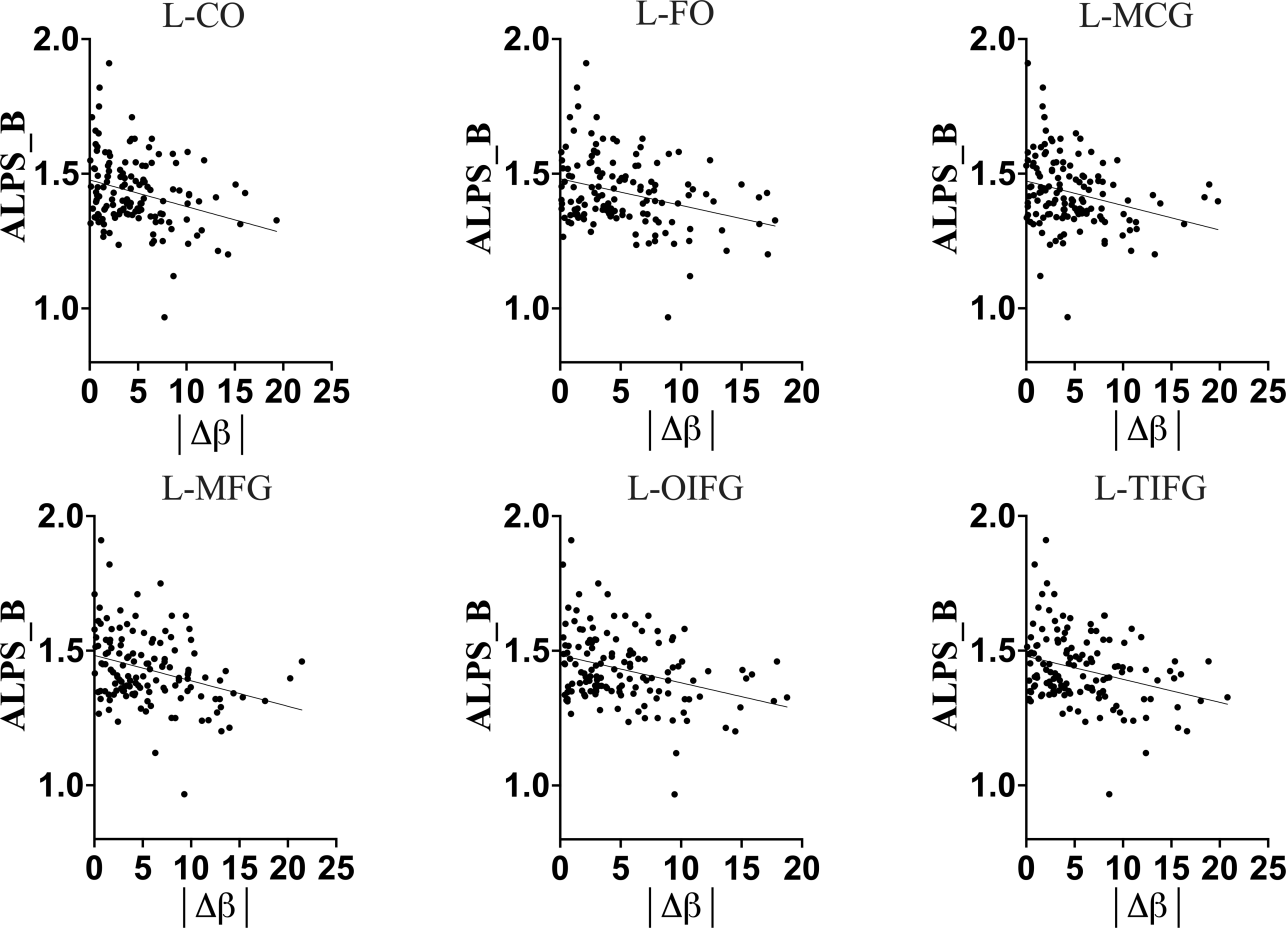
Full-Sample association of DTI-ALPS index of glymphatic clearance and brain-predicted age difference (brain-PAD) of brain structures. The greater brain-PADs was associated with a lower DTI-ALPS index, suggesting that in general population an increasing deviation of predicted age from chronological age is associated with a decline in glymphatic clearance. L-CO, Left central operculum; L-FO, Left frontal operculum; L-MCG, Left middle cingulate gyrus; L-MFG, Left middle frontal gyrus; L-OIFG, Left opercular inf. frontal gyrus; L-TIFG, Left triangular inf. frontal gyrus.

First, we observed that in the general population (HC+HIV), the DTI-ALPS index exhibited a strong correlation with chronological age (r = -0.2685, p = 0.0019, **Table 2**), global biological age of the brain (**Figure 2**; **Table 2**), predicted ages of brain structures (**Figure 2**; **Table 2**), and brain-predicted age differences (brain-PADs) of the brain structures (**Figure 3**).

**Table 2:** Relationship of brain-predicted age and glymphatic performance.

Brain region	All data		HC		HIV	
	*r*	*p*	*r*	*p*	*r*	*p*
Chronological age	-0.2685	0.0019	-0.4048	0.0064	-0.1637	0.1170
Whole brain	-0.2361	0.0064	-0.3208	0.0337	-0.1093	0.2968
External CSF	-0.2636	0.0023	-0.2841	0.0616	-0.1624	0.1198
3rd ventricle	-0.1945	0.0255	-0.2492	0.1029	-0.0848	0.4192
4th ventricle	-0.2802	0.0011	-0.3919	0.0085	-0.1000	0.3404
Right accumbens	-0.1835	0.0351	-0.1963	0.2017	-0.1037	0.3227
Left accumbens	-0.2015	0.0205	-0.2017	0.1892	-0.1276	0.2228
Right amygdala	-0.2260	0.0092	-0.3178	0.0355	-0.0979	0.3503
Left amygdala	-0.2231	0.0101	-0.3031	0.0455	-0.0935	0.3729
Brainstem	-0.2640	0.0022	-0.3833	0.0102	-0.0999	0.3406
Right caudate	-0.1905	0.0287	-0.1774	0.2494	-0.1171	0.2636
Left caudate	-0.2132	0.0141	-0.1593	0.3018	-0.1648	0.1144
Right cerebellum exterior	-0.2992	0.0005	-0.3959	0.0078	-0.1199	0.2525
Left cerebellum exterior	-0.2832	0.0010	-0.3795	0.0111	-0.1112	0.2887
Right cerebellum White Matter	-0.2904	0.0007	-0.4031	0.0067	-0.1113	0.2884
Left cerebellum White Matter	-0.2854	0.0009	-0.4089	0.0059	-0.0943	0.3686
Right cerebral White Matter	-0.2340	0.0069	-0.2736	0.0724	-0.1326	0.2051
Left cerebral White Matter	-0.2369	0.0062	-0.2079	0.1757	-0.1704	0.1025
Right hippocampus	-0.2415	0.0053	-0.3494	0.0201	-0.1027	0.3272
Left hippocampus	-0.2415	0.0053	-0.3278	0.0298	-0.0991	0.3445
Right inf. lateral ventricle	-0.2502	0.0038	-0.3480	0.0206	-0.1195	0.2540
Left inf. lateral ventricle	-0.2294	0.0081	-0.2967	0.0505	-0.1036	0.3229
Right lateral ventricle	-0.2117	0.0148	-0.2492	0.1029	-0.1104	0.2920
Left lateral ventricle	-0.2204	0.0111	-0.2210	0.1494	-0.1363	0.1927
Right pallidum	-0.1888	0.0302	-0.2208	0.1499	-0.0953	0.3633
Left pallidum	-0.2128	0.0143	-0.2204	0.1505	-0.1299	0.2144
Right putamen	-0.2086	0.0164	-0.2494	0.1025	-0.1135	0.2789
Left putamen	-0.2234	0.0100	-0.2213	0.1489	-0.1485	0.1553
Right thalamus	-0.1748	0.0450	-0.2245	0.1429	-0.0698	0.5059
Left thalamus	-0.2045	0.0186	-0.2355	0.1239	-0.1037	0.3224
Right ventral DC	-0.2086	0.0164	-0.2991	0.0485	-0.0811	0.4395
Left ventral DC	-0.2258	0.0092	-0.3114	0.0396	-0.0930	0.3755
Lobules I-V	-0.2603	0.0026	-0.3877	0.0093	-0.0804	0.4434
Lobules VI-VII	-0.2931	0.0006	-0.4238	0.0041	-0.0920	0.3807
Lobules VIII-X	-0.3037	0.0004	-0.4211	0.0044	-0.1079	0.3032
Left basal forebrain	-0.2001	0.0214	-0.2436	0.1111	-0.1030	0.3259
Right basal forebrain	-0.2006	0.0211	-0.2513	0.0999	-0.1021	0.3301
Right anterior cingulate gyrus	-0.2085	0.0165	-0.1792	0.2444	-0.1506	0.1496
Left anterior cingulate gyrus	-0.2181	0.0120	-0.1623	0.2926	-0.1771	0.0895
Right anterior insula	-0.2313	0.0076	-0.2954	0.0515	-0.1265	0.2271
Left anterior insula	-0.2318	0.0075	-0.2329	0.1281	-0.1640	0.1163
Right anterior orbital gyrus	-0.1817	0.0371	-0.1986	0.1963	-0.0998	0.3412
Left anterior orbital gyrus	-0.1790	0.0400	-0.1571	0.3083	-0.1392	0.1834
Right angular gyrus	-0.2203	0.0112	-0.2616	0.0863	-0.1244	0.2347
Left angular gyrus	-0.2006	0.0211	-0.0901	0.5608	-0.1788	0.0864
Right calcarine cortex	-0.2251	0.0095	-0.3350	0.0262	-0.0760	0.4689
Left calcarine cortex	-0.2313	0.0076	-0.3367	0.0254	-0.0787	0.4533
Right central operculum	-0.2264	0.0090	-0.2968	0.0504	-0.1266	0.2266
Left central operculum	-0.2323	0.0074	-0.2147	0.1617	-0.1745	0.0944
Right cuneus	-0.2165	0.0127	-0.2775	0.0682	-0.0905	0.3882
Left cuneus	-0.2200	0.0113	-0.2474	0.1055	-0.1116	0.2869
Right entorhinal area	-0.2199	0.0113	-0.3077	0.0421	-0.0997	0.3415
Left entorhinal area	-0.2116	0.0149	-0.2837	0.0620	-0.0927	0.3771
Right frontal operculum	-0.2312	0.0077	-0.2946	0.0522	-0.1266	0.2265
Left frontal operculum	-0.2434	0.0049	-0.2322	0.1293	-0.1860	0.0743
Right frontal pole	-0.1406	0.1077	-0.0661	0.6699	-0.1183	0.2587
Left frontal pole	-0.1612	0.0647	-0.0530	0.7325	-0.1665	0.1106
Right fusiform gyrus	-0.2627	0.0023	-0.3615	0.0159	-0.1111	0.2892
Left fusiform gyrus	-0.2562	0.0030	-0.3341	0.0266	-0.1027	0.3271
Right gyrus rectus	-0.1767	0.0427	-0.1935	0.2083	-0.0997	0.3416
Left gyrus rectus	-0.1827	0.0360	-0.1993	0.1946	-0.1100	0.2937
Right inf. occipital gyrus	-0.2378	0.0060	-0.3146	0.0375	-0.1076	0.3046
Left inf. occipital gyrus	-0.2374	0.0061	-0.3110	0.0399	-0.1060	0.3121
Right inf. temporal gyrus	-0.2678	0.0019	-0.3649	0.0149	-0.1218	0.2447
Left inf. temporal gyrus	-0.2274	0.0087	-0.2815	0.0641	-0.0979	0.3507
Right lingual gyrus	-0.2404	0.0055	-0.3605	0.0162	-0.0793	0.4498
Left lingual gyrus	-0.2512	0.0037	-0.3775	0.0115	-0.0776	0.4596
Right lateral orbital gyrus	-0.2144	0.0136	-0.2784	0.0672	-0.1105	0.2916
Left lateral orbital gyrus	-0.1846	0.0341	-0.1826	0.2355	-0.1374	0.1890
Right middle cingulate gyrus	-0.2068	0.0174	-0.1673	0.2776	-0.1456	0.1639
Left middle cingulate gyrus	-0.2135	0.0140	-0.1524	0.3232	-0.1657	0.1124
Right medial frontal cortex	-0.1929	0.0267	-0.2108	0.1695	-0.1110	0.2893
Left medial frontal cortex	-0.2000	0.0215	-0.1982	0.1972	-0.1363	0.1928
Right middle frontal gyrus	-0.2247	0.0096	-0.2251	0.1417	-0.1583	0.1295
Left middle frontal gyrus	-0.2360	0.0064	-0.1394	0.3667	-0.2351	0.0233
Right middle occipital gyrus	-0.2259	0.0092	-0.2733	0.0727	-0.1238	0.2373
Left middle occipital gyrus	-0.1840	0.0347	-0.1270	0.4113	-0.1324	0.2060
Right medial orbital gyrus	-0.1848	0.0339	-0.2128	0.1655	-0.1003	0.3388
Left medial orbital gyrus	-0.1822	0.0365	-0.1989	0.1954	-0.1128	0.2816
Right postcentral gyrus medial segment	-0.2620	0.0024	-0.2627	0.0850	-0.1626	0.1194
Left postcentral gyrus medial segment	-0.2741	0.0015	-0.2205	0.1503	-0.2125	0.0409
Right precentral gyrus medial segment	-0.2464	0.0044	-0.2366	0.1220	-0.1633	0.1177
Left precentral gyrus medial segment	-0.2548	0.0032	-0.2102	0.1708	-0.1932	0.0635
Right sup. frontal gyrus medial segment	-0.1923	0.0272	-0.1174	0.4478	-0.1601	0.1254
Left sup. frontal gyrus medial segment	-0.2097	0.0158	-0.1070	0.4894	-0.1978	0.0574
Right middle temporal gyrus	-0.2591	0.0027	-0.3726	0.0127	-0.1261	0.2282
Left middle temporal gyrus	-0.2211	0.0108	-0.2547	0.0952	-0.1194	0.2544
Right occipital pole	-0.2633	0.0023	-0.3316	0.0279	-0.1232	0.2392
Left occipital pole	-0.2664	0.0020	-0.3487	0.0203	-0.1232	0.2395
Right occipital fusiform gyrus	-0.2733	0.0015	-0.3700	0.0134	-0.1134	0.2791
Left occipital fusiform gyrus	-0.2718	0.0016	-0.3705	0.0133	-0.1037	0.3227
Right opercular inf. frontal gyrus	-0.2391	0.0058	-0.2783	0.0674	-0.1565	0.1341
Left opercular inf. frontal gyrus	-0.2479	0.0042	-0.2090	0.1734	-0.2111	0.0423
Right orbital inf. frontal gyrus	-0.2187	0.0118	-0.2759	0.0699	-0.1179	0.2605
Left orbital inf. frontal gyrus	-0.2034	0.0193	-0.2022	0.1880	-0.1467	0.1607
Right posterior cingulate gyrus	-0.2134	0.0140	-0.2644	0.0829	-0.0936	0.3723
Left posterior cingulate gyrus	-0.2165	0.0127	-0.2494	0.1025	-0.1079	0.3031
Right precuneus	-0.2307	0.0078	-0.2771	0.0686	-0.1077	0.3043
Left precuneus	-0.2361	0.0064	-0.2439	0.1105	-0.1384	0.1859
Right parahippocampal gyrus	-0.2444	0.0047	-0.3514	0.0193	-0.1004	0.3382
Left parahippocampal gyrus	-0.2505	0.0038	-0.3482	0.0205	-0.0990	0.3451
Right posterior insula	-0.2273	0.0088	-0.3172	0.0359	-0.1120	0.2850
Left posterior insula	-0.2322	0.0074	-0.2538	0.0965	-0.1453	0.1646
Right parietal operculum	-0.2220	0.0105	-0.2764	0.0693	-0.1319	0.2075
Left parietal operculum	-0.2063	0.0176	-0.1234	0.4250	-0.1748	0.0938
Right postcentral gyrus	-0.2370	0.0062	-0.2605	0.0876	-0.1520	0.1458
Left postcentral gyrus	-0.2494	0.0039	-0.1480	0.3378	-0.2307	0.0261
Right posterior orbital gyrus	-0.2210	0.0109	-0.2870	0.0589	-0.1166	0.2658
Left posterior orbital gyrus	-0.1985	0.0225	-0.2317	0.1302	-0.1175	0.2619
Right planum polare	-0.2397	0.0056	-0.3305	0.0285	-0.1215	0.2459
Left planum polare	-0.2320	0.0074	-0.2549	0.0950	-0.1467	0.1605
Right precentral gyrus	-0.2304	0.0079	-0.2488	0.1035	-0.1540	0.1405
Left precentral gyrus	-0.2443	0.0048	-0.1483	0.3367	-0.2308	0.0260
Right planum temporale	-0.2453	0.0046	-0.3247	0.0315	-0.1475	0.1582
Left planum temporale	-0.2168	0.0125	-0.1877	0.2225	-0.1579	0.1306
Right subcallosal area	-0.2021	0.0202	-0.2462	0.1072	-0.1075	0.3052
Left subcallosal area	-0.2040	0.0190	-0.2447	0.1095	-0.1097	0.2951
Right sup. frontal gyrus	-0.2075	0.0170	-0.1364	0.3774	-0.1757	0.0920
Left sup. frontal gyrus	-0.2219	0.0105	-0.0941	0.5437	-0.2277	0.0281
Right supplementary motor cortex	-0.2166	0.0126	-0.1605	0.2981	-0.1716	0.1000
Left supplementary motor cortex	-0.2258	0.0092	-0.1369	0.3756	-0.2013	0.0530
Right supramarginal gyrus	-0.2323	0.0074	-0.2732	0.0727	-0.1473	0.1588
Left supramarginal gyrus	-0.2035	0.0193	-0.0836	0.5897	-0.1916	0.0658
Right sup. occipital gyrus	-0.2463	0.0044	-0.2943	0.0525	-0.1283	0.2203
Left sup. occipital gyrus	-0.2133	0.0140	-0.2050	0.1820	-0.1300	0.2144
Right sup. parietal lobule	-0.2507	0.0037	-0.2777	0.0680	-0.1431	0.1712
Left sup. parietal lobule	-0.2555	0.0031	-0.1848	0.2297	-0.2099	0.0435
Right sup. temporal gyrus	-0.2532	0.0034	-0.3664	0.0144	-0.1336	0.2017
Left sup. temporal gyrus	-0.2295	0.0081	-0.2521	0.0988	-0.1413	0.1766
Right temporal pole	-0.2367	0.0063	-0.3216	0.0333	-0.1195	0.2538
Left temporal pole	-0.1917	0.0276	-0.2408	0.1154	-0.0971	0.3546
Right triangular inf. frontal gyrus	-0.2201	0.0112	-0.2657	0.0813	-0.1285	0.2196
Left triangular inf. frontal gyrus	-0.2398	0.0056	-0.2085	0.1743	-0.2030	0.0510
Right transverse temporal gyrus	-0.2253	0.0094	-0.3083	0.0418	-0.1177	0.2610
Left transverse temporal gyrus	-0.2257	0.0093	-0.2259	0.1404	-0.1493	0.1531

r, Pearson’s correlation coefficient; p, p-value; HC, heathy controls; HIV, subjects with HIV.

The older chronological age, along with increased predicted age and brain-PAD of brain structures, were associated with a lower DTI-ALPS index, suggesting that typical normal aging is accompanied by declining glymphatic clearance in the general population. These findings corroborate the earlier findings indicating a negative relationship between aging and glymphatic performance in general population.

**Figure 4:**
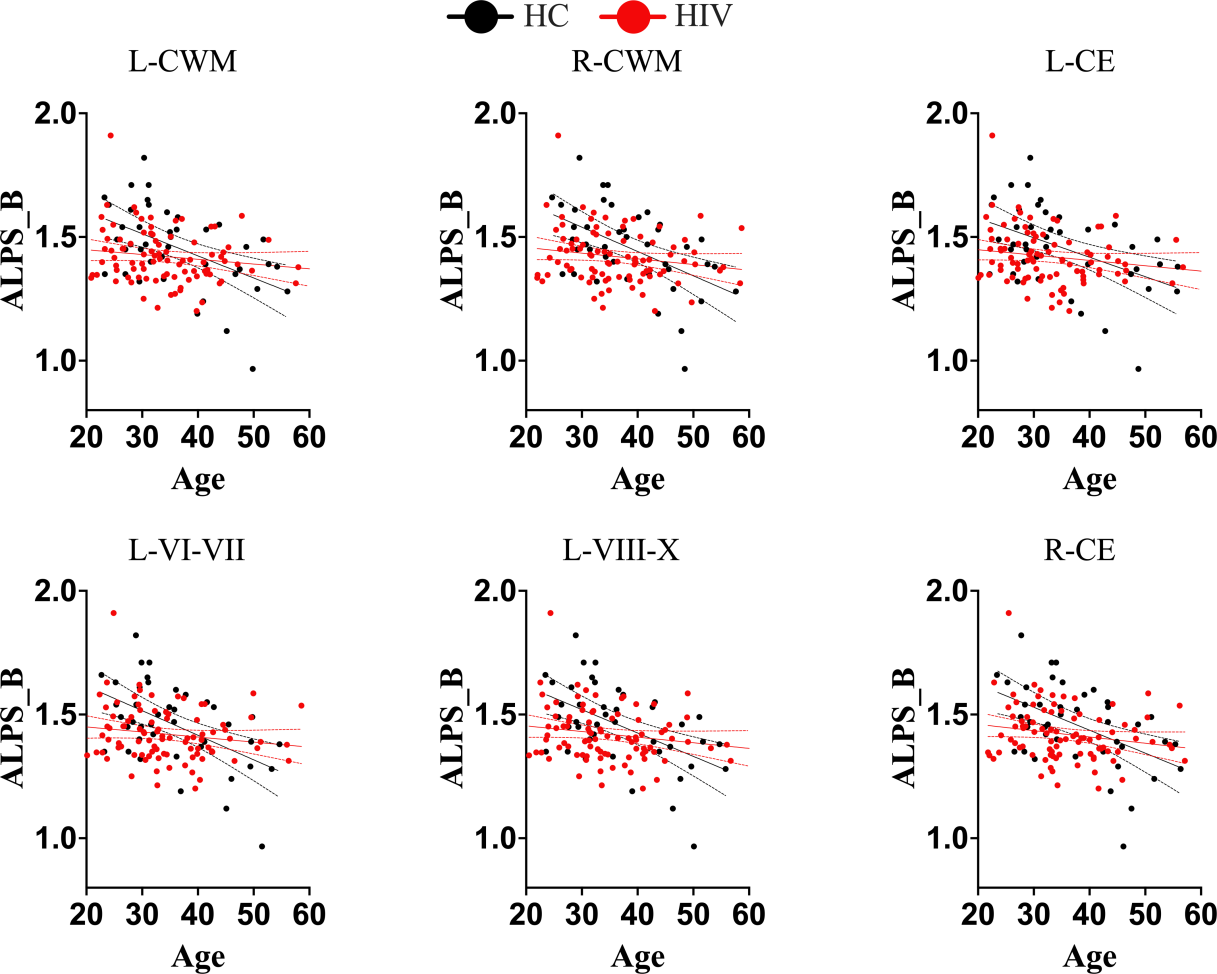
Trajectories of DTI-ALPS index of glymphatic clearance and predicted ages of brain structures for separate groups (healthy controls and subjects with HIV). While normal subjects maintained the trajectory of glymphatic clearance and brain aging, as there was strong correlation between glymphatic clearance and predicted ages of brain structures, subjects with HIV lost the trajectory. There was no significant correlations between the glymphatic clearance and predicted ages of brain structures in subjects with HIV. These findings suggest that HIV is likely to disrupt the trajectory of glymphatic clearance with brain aging. L-CWM, left cerebellum white matter; R-CWM, Right cerebellum white matter; L-CE, left cerebellum exterior; L-VI-VII, Lobules VI-VII; L-VIII-X, lobules VIII-X; R-CE, right cerebellum exterior.

**Figure 5:**
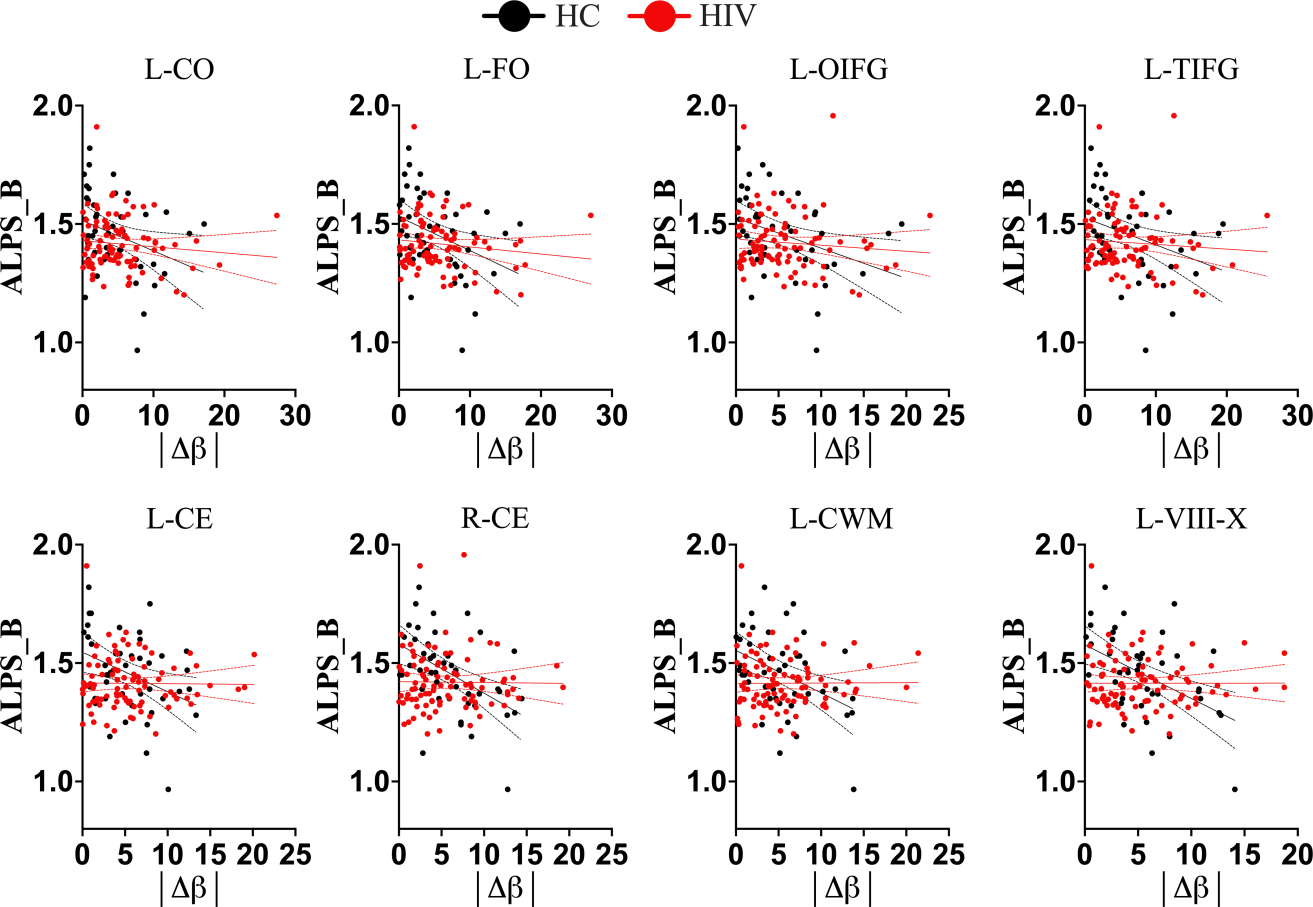
Trajectories of Brain-Predicted Age Difference (brain-PAD) and DTI-ALPS index in different conditions. In healthy controls (*black color*), there is a clear trajectory of glymphatic clearance and brain-PADs of several brain structures, as displayed by strong correlations between them. However, in subjects with HIV(*red color*), the normal trajectory between the two aspects is lost, suggesting that HIV effects on glymphatic clearance manifest as early as even when accelerated brain aging (or underlying structural pathology) is subtle. L-CO, left central operculum; L-FO, left frontal operculum; L-OIFG, left opercular inf. frontal gyrus; L-TIFG, left triangular inf. frontal gyrus; L-CE, left cerebellum exterior; R-CE, right cerebellum exterior; L-CerelellumWM, left cerebellum White Matter; L-VII-X, lobules VIII-X; |Δβ|, absolute difference between predicted age of brain structure and chronological age.

We conducted further analyses to determine whether HIV infection has an independent impact or induces disruptions to the typical trajectory of glymphatic clearance in the aging brain, contrary to those observed in healthy subjects. We found that while healthy controls maintained/retained strong relationships between the DTI-ALPS index and aging indices, as seen in the general population, those infected with HIV showed disrupted relationships (**Table 2**). To be more specific, the DTI-ALPS index in healthy subjects exhibited a strong relationships with chronological age (**Table 2**, HC: r = -0.4048, p = 0.0064; HIV: r = -0.1637, p = 0.1170), predicted age of brain structures (**Table 2**; **Figure 4**), and brain-predicted age differences (**Figure 5**). With predicted ages of brain structures (**Table 2**; **Figure 4**), the most evident relationships of DTI-ALPS were in the 4^th^ ventricle, right/left cerebellum exterior, right/left cerebellum white matter, and lobules (I-IV, VI-VII, VIII-X) (**Table 2**). With brain predicted age differences (**Figure 5**), such relationships were mostly identified in the structures of the left central operculum (r = -0.4327, p = 0.0038), left frontal operculum (r = -0.4689, p = 0.0015), left opercular inf. frontal gyrus (r = -0.4639, p = 0.0017), and left triangular inf. frontal gyrus (r = -0.4089, p = 0.0065), as well as in the right cerebellum exterior (r = -0.4036, p = 0.0073), left cerebellum exterior (r = -0.472, p = 0.0014), left cerebellum White Matter (r = -0.4247, p = 0.0045), lobules VI-VII (r = -0.4577, p = 0.002), lobules VIII-X (r = -0.4831, p = 0.001), left occipital fusiform gyrus (r = -0.4084, p = 0.0066), and left posterior insula (r = -0.3935, p = 0.009). These relationships observed in healthy controls were similar to those seen in the general population (**Table 2**; **Figures 2**, **3**). However, none of these significant relationships were found in subjects with HIV, suggesting that HIV attenuates the existing relationships between glymphatic function and proxies of brain aging, likely due to ongoing pathology induced by HIV, independent of age. We conducted slope test analyses to assess the significance of the differences between the slopes, testing whether the trends of the relationships between the DTI-ALPS glymphatic index and brain-PADs were indeed different between the two groups (HC versus HIV). Our results revealed that the two groups exhibited different trends in the relationship between the DTI-ALPS glymphatic index and brain-PADs (**Figure 5**, see **Table 3** for more details).

**Table 3:** Brain-predicted age differences and glymphatic performance (slopes comparisons).

Brain region	HC	HIV	t	*P*-value
4th ventricle	-0.0403	0.0028	-2.5067	0.0134
Right cerebellum exterior	-0.0512	0.0065	-3.8169	0.0002
Left cerebellum exterior	-0.0432	0.0016	-2.8614	0.0049
Right cerebellum White Matter	-0.0374	0.0034	-2.3927	0.0181
Left cerebellum White Matter	-0.0363	0.0029	-2.6215	0.0097
Left cerebral White Matter	-0.0373	-0.0030	-2.0899	0.0385
Lobules I-V	-0.0407	0.0094	-3.1131	0.0023
Lobules VI-VII	-0.0571	-0.0033	-3.0261	0.0030
Lobules VIII-X	-0.0529	0.0029	-3.3432	0.0011
Left calcarine cortex	-0.0370	0.0099	-2.5647	0.0114
Left central operculum	-0.0401	-0.0021	-2.4417	0.0159
Left frontal operculum	-0.0322	-0.0015	-2.1024	0.0374
Left fusiform gyrus	-0.0372	0.0022	-2.0883	0.0386
Left inf. occipital gyrus	-0.0398	0.0065	-2.8916	0.0045
Left inf. temporal gyrus	-0.0319	0.0039	-2.1867	0.0305
Left lingual gyrus	-0.0383	0.0015	-2.5622	0.0115
Left medial frontal cortex	0.0334	-0.0034	2.5249	0.0127
Left occipital fusiform gyrus	-0.0467	-0.0047	-2.3995	0.0178
Left opercular inf. frontal gyrus	-0.0476	-0.0073	-2.5110	0.0132
Right postcentral gyrus	0.0250	-0.0091	2.1369	0.0344
Right precentral gyrus	0.0471	-0.0129	3.4790	<0.0007
Left sup. temporal gyrus	-0.0261	0.0075	-2.6002	0.0103
Right triangular inf. frontal gyrus	0.0195	-0.0084	2.3207	0.0218
Left triangular inf. frontal gyrus	-0.0481	-0.0036	-2.7376	0.0070
Left transverse temporal gyrus	-0.0292	-0.0008	-2.0074	0.0468

*The values in HC and HIV groups are the slopes for the lines fitting glymphatic clearance proxy measure and brain-predicted age difference (brain-PAD). The regions shown in this table are only those exhibiting significant differences in their slopes between HC and HIV; t and p are the statistical values for the test.

### 3.3 The link between neuroimaging markers and clinical outcomes

**Figure 6:**
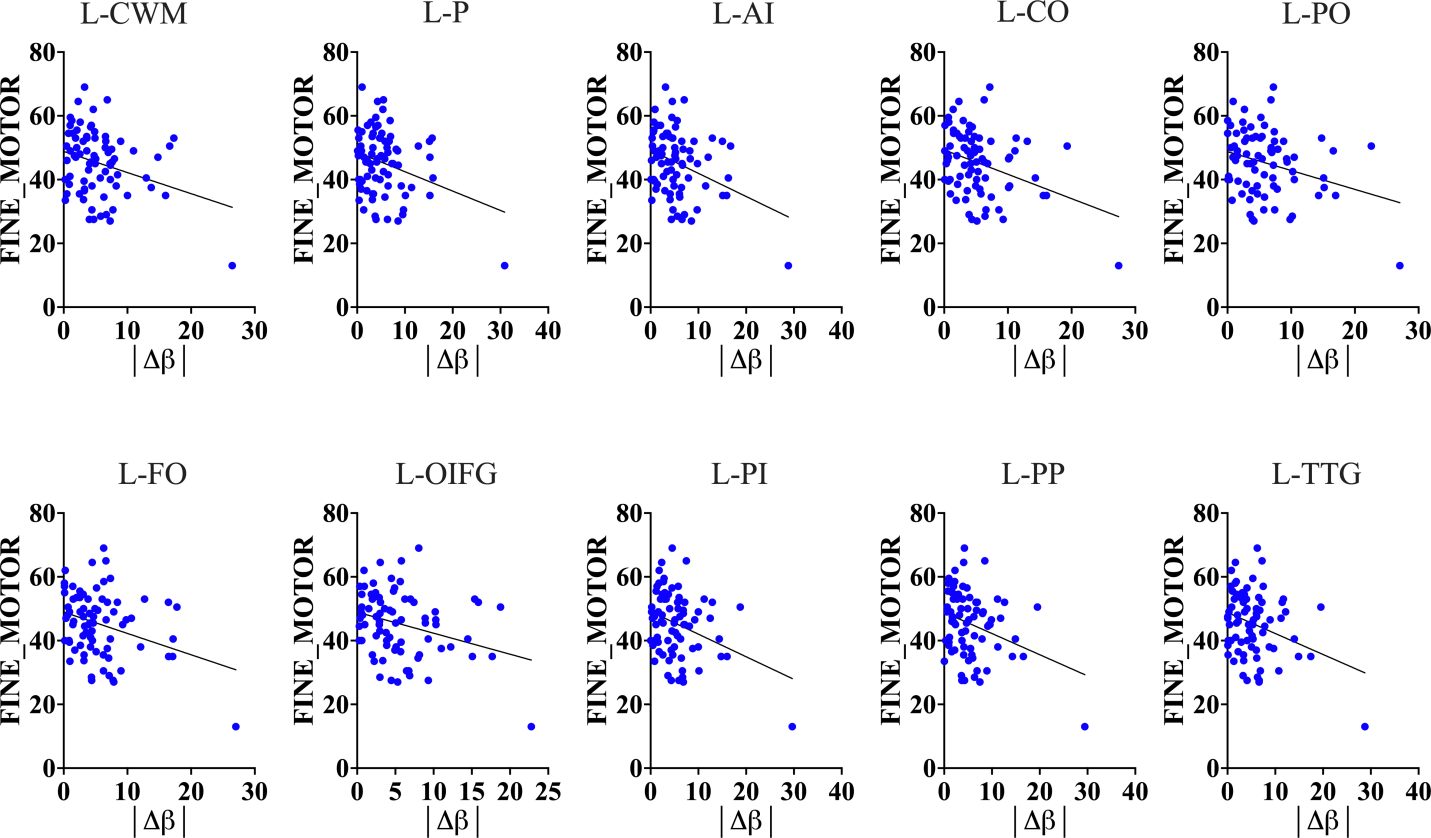
Motoric function and Brain-Predicted Age Difference (brain-PAD). Results show that brain-predicted age difference of several brain structures is a good predictor of motoric and executive performance (see **Supplementary Table S3** for executive performance and brain-PAD).The results suggest that increased brain-PAD (reflecting underlying neuropathology) can explain a decline in fine motor and executive functions often seen in HIV patients. L-CWM, Left cerebral White Matter; L-P, Left putamen; L-AI, Left anterior insula; L-CO, Left central operculum; L-PO, Left parietal operculum; L-FO, Left frontal operculum; L-OIFG, Left opercular inf. frontal gyrus; L-PI, Left posterior insula; L-PP, Left planum polare; L-TTG, Left transverse temporal gyrus; |Δβ|, absolute difference between predicted age of brain structure and chronological age.

To test whether the impact of HIV on accelerated brain-aging and glymphatic dysfunction is linked to or exacerbates cognitive impairment in HIV infection, we conducted analyses of the association between brain-PADs and cognitive functions across six domains. Our results demonstrated that a greater brain-PAD is significantly related to lower performance in fine motor function (**Figure 6**, also see **Supplementary Table S3**). These findings suggest that the impact of HIV on brain-PADs and glymphatic dysfunction is linked to impairment in motor function. On the other hand, these findings underscore that brain-PADs conceal relevant information related to underlying pathological conditions in brain structures following infection or disease. In our case, several brain structures exhibited strong association between their increased brain-PADs and reduced motoric function. These structures include left cerebral White Matter (r = -0.2987, p = 0.0083), left putamen (r = -0.2935, p = 0.0096), left anterior insula (r = -0.3452, p = 0.0021), left central operculum (r = -0.3549, p = 0.0015), left frontal operculum (r = -0.3211, p = 0.0044), left opercular inf. frontal gyrus (r = -0.3061, p = 0.0068), left orbital inf. frontal gyrus (r = -0.3039, p = 0.0072), left posterior insula (r = -0.3336, p = 0.003), left parietal operculum (r = -0.2966, p = 0.0088), left planum polare (r = -0.3182, p = 0.0048), and left transverse temporal gyrus (r = -0.3137, p = 0.0055). Our results also showed that increased brain-PADs in some regions, particularly those where increased brain aging is highly associated with reduced glymphatic performance, were linked to lower performance in executive memory function (see **Supplementary Table S3**). These findings underscore that HIV pathology in both the brain aging process and glymphatic clearance function may expedite the impairment of other cognitive domains, such as the executive function domain.

Surprisingly, greater brain-PAD in other brain regions—especially those in the right hemisphere—exhibited a strong association with higher performance in language and working memory. We ascribe this phenomenon to compensatory mechanisms of the brain, which may likely occur to adjust for the underlying pathology. The brain regions whose brain-PADs exhibit this feature with functional performance of language include the right superior frontal gyrus (p = 0.0031) and the right supplementary motor cortex (p = 0.0046); whereas those exhibiting this feature with working memory include the postcentral gyrus, superior frontal gyrus, supramarginal gyrus, and superior parietal lobule. Our study did not disclose any other cognitive domains exhibiting an association with brain-PADs besides the ones mentioned above.

## 4 Discussion

### 4.1 Glymphatic system and HIV

In this study, we investigated the impact of HIV on brain aging and the glymphatic profile. Specifically, we examined whether HIV infection accelerates brain aging and induces disruptions in the glymphatic clearance profile or trajectory observed in the aging process. We further assessed whether accelerated brain aging and glymphatic impairment complement each other and whether their influence exacerbates pathological conditions in other domains of cognitive function. We used brain-PAD and the DTI-ALPS index as proxy measures of brain aging and glymphatic clearance function, respectively. Their use in this study was part of initiatives to validate their utility in reflecting ongoing or underlying neuropathology following the invasion of the CNS by infection or disease.

Our results demonstrated that the invasion of the CNS by HIV disrupts the glymphatic clearance profile/trajectory normally observed with aging. This was evidenced by the disappearance of the typical relationship between the glymphatic index (DTI-ALPS) and aging indices in subjects with HIV. While this was evident in subjects with HIV, those not affected by HIV (healthy subjects) retained a typical trajectory similar to that seen in the general population. The healthy subjects had a glymphatic profile characterized by greater performance (high DTI-ALPS) at a young age and lower performance (low DTI-ALPS) at an old age, consistent with previous studies. However, for HIV subjects, the glymphatic clearance function was impaired regardless of age (see **Supplementary Table S2**). The glymphatic clearance function of younger subjects was not spared (significantly altered) in HIV infection. Both the younger and the older subjects with HIV had relatively similar lower performance (low DTI-ALPS). This HIV-induced disruption affected the typical relationship of glymphatic function with age-both chronological age and brain-predicted age.

Our analysis of brain-PADs demonstrated the role of accelerated brain aging as a surrogate biomarker of the underlying neuropathology following an infection or disease. We specifically observed that the HIV entry to the brain induces changes that accelerate brain aging. This is evidenced by greater brain-PADs detected in subjects with HIV in several brain structures compared to healthy controls. Among the regions exhibiting accelerated aging, the following appeared to age faster: the postcentral gyrus, precentral gyrus, precuneus, parietal operculum, supramarginal gyrus, superior occipital gyrus and occipital pole, middle frontal gyrus, and angular gyrus. These findings suggest that HIV accelerates brain aging and validate the utility of increased brain-PADs as indicative of underlying neurobiological mechanisms affecting brain health, consistent with other previous works (31, 32).

Our analyses also showed that HIV attenuates the relationship between the glymphatic index and brain-aging indices. In fact, while the general population (HC+HIV) had a glymphatic index (DTI-ALPS) associated with predicted ages and brain-PADs of brain structures, similar to healthy subjects, this relationship was lost in the HIV group. Further analyses testing for the significance of differences in the slopes/trends of these patterns of relationships exhibited by DTI-ALPS and brain-PADs confirmed that the two groups(HIV versus HC) indeed exhibited different trends in the relationship. Interestingly, the same regions whose brain-PADs’ relationship with DTI-ALPS were altered displayed a stronger association with cognitive performance of motor function, and abstract and executive function. In particular, greater brain-PADs was associated with lower performance in these cognitive domains. These findings suggest that while the entry of HIV into the CNS alters the glymphatic system and the brain’s aging process, these alterations, as evidenced by DTI-ALPS and brain-PADs, may be a reflection of the ongoing HIV pathology in cognitive functions, particularly those involving motor and executive domains. The same underlying pathology may be responsible for the attenuation of the standard trajectory of glymphatic performance in aging brain. These findings align with other neuroimaging-based studies that demonstrate the link between accelerated brain aging and deficits in key cognitive domains such as executive function, attention, working memory, and information processing speed (16, 33).

Our data also revealed aspects of hemispheric asymmetry in the changes induced by HIV pathology. The increased brain-PADs, indicative of accelerated aging in HIV patients, were more evident in the left brain. Since all subjects were right-handed with left-brain dominance, these findings may suggest that HIV alterations or neuropathology begin in the dominant brain (the left brain in this case) and propagate to the right as the disease advances or in later stages. This explanation and findings align with previous works identifying the left brain as more vulnerable to inflammatory neurological conditions such as PD (34–36) and AIDS (37, 38). Some works expand the discussion, demonstrating that although the brain is inherently hemispherically asymmetric even in normal individuals, the presence of inflammatory neurological complications such as HIV pathology or PD may intensify this characteristic (36, 39).

From the core aspect of this study, we see that several brain structures experienced HIV impacts, either in their aging patterns or glymphatic profile/trajectory with aging indices (brain-PAD). Diving in-depth into these structures, we see that some of these structures have been implicated in HIV pathology previously through different types of analyses. For example, a study by Tangliati et al. (40) reported focal degeneration of the cerebellar granular cell layer and prominent cerebellar atrophy in the neuroimaging data of HIV-infected subjects (40). The alterations within the cerebellar morphometry or age difference are believed to underlie cognitive and motor declines (41–43). The left postcentral gyrus medial segment, which showed greater brain-PAD for subjects with HIV in our study, was also implicated in HIV pathology in the study by Casagrande et al. (37). The authors detected strong spontaneous cortical activity in this area and reduced cortical gray matter thickness. Another study implicated the frontal operculum in the dysfunction of memory and learning ability in individuals with HIV who developed HAND, in a study of functional connectivity. In that study, the frontal operculum’s functional connectivity with the right superior frontal gyrus was specifically decreased. We found similar patterns in our study, where the frontal and central operculum had significantly increased brain-PAD in subjects with HIV. Coincidentally, the brain-PADs of these regions exhibited a different trend in their relationship with glymphatic clearance in HIV infection compared to those in normal conditions. On the other hand, their increased brain-PADs appeared to contribute significantly to cognitive dysfunction in motor and executive domains.

The findings from this study identify an abnormal trend in the relationship between glymphatic function and brain aging proxies as a potential biomarker of HIV pathology, which may be involved in the motor and executive impairments often seen in subjects with HIV. Limited studies have identified brain alterations underlying motor and executive dysfunction (44–47). Considering the complexity of the underlying processes for executing tasks in these domains (48–50), it is clear that brain alterations involved in their dysfunction must involve more complex biological aspects, particularly those involving the complex interaction of viral activity, glymphatic and aging processes (51, 52), rather than one aspect. Thus, this study offers new insights into another dimension of HIV pathology and possible mechanisms involved in changes to motor and executive function. In HIV, several mechanisms can disrupt the normal functioning of the glymphatic system (53) and alter its relationship with age. One possible mechanism is through HIV compromising the integrity of the BBB, leading to increased permeability, which further results in an excessive influx of inflammatory cytokines and harmful substances in the brain parenchyma (54), exacerbating neuroinflammation and impairing glymphatic drainage. The inflammation resulting from immune responses and the presence of viral particles damages neural tissues and impairs the function of glial cells essential for glymphatic function (55–57). These HIV-induced inflammatory processes/activity may lead to decreased expression or mislocalization of aquaporin-4 (AQP4) from astrocytic end feet, resulting in decreased interstitial flow, promoting accumulation of extracellular waste products like hyperphosphorylated Tau (12). Immunocompromised states of the brain trigger accelerated brain aging and induce age-related impairment of the glymphatic system due to additional stressors such as chronic inflammation or opportunistic infections that are more prevalent in HIV. Sleep disturbances experienced by individuals with HIV due to factors such as depression, anxiety, or side effects from antiretroviral therapy further reduce the clearance of neurotoxic waste products from the brain (58, 59), eventually affecting its normal trajectory over time. Conditions that are comorbid with HIV, such as cardiovascular diseases or metabolic syndrome (60), may further impair glymphatic function through vascular disturbances and by impairing the perfusion pressure necessary for optimal CSF dynamics.

Other influencing factors: Our findings also revealed that ART status, particularly the suppressive effects of ART, did not influence the outcomes of our main findings. There were no significant differences in either brain-PADs or glymphatic index between those with successful viral suppression and those where viral suppression was not effective. The duration of HIV did not seem to impact brain-PAD or glymphatic clearance function in our data. There was no link or association (at least for this data) between the duration of HIV and these two aspects of biology. Nevertheless, our data revealed that a compromised immune status may impact our findings. A reduction in the CD4/CD8 ratio due to HIV infection was associated with increased brain aging (greater brain-PADs) in some structures, particularly the left amygdala, brainstem, left hippocampus, and left ventral DC. The HAND status did not appear to influence glymphatic clearance outcomes but had a significant impact on the brain-PAD of external CSF (HAND-: 1.203, HAND+: 4.861, T = 4.239, P = 0.000084), suggesting that individuals with HAND experienced faster aging in the structures accommodating CSF than those without HAND.

### 4.2 Limitations

In this study, we employed two approaches to mitigate or observe the effects of potential confounders or variables in the study. First, we excluded subjects with characteristics such as signs of neurological disorders, brain injury, brain lesions, cerebral atrophy, or illicit drug or alcohol use. Second, we analyzed and observed how other factors, such as ART status and duration of HIV, impacted the findings. However, we could not investigate how systemic inflammation levels impact the findings; thus, future studies are warranted to further explore how these levels could influence glymphatic clearance and brain aging.

## 5 Conclusion

In this study, we demonstrate that HIV entry to the CNS alters glymphatic clearance function and accelerates brain aging [as indicated through brain-predicted age differences (brain-PAD)] of several structures. The alteration of glymphatic clearance function disrupts its trajectory in the aging brain through HIV-induced inflammatory processes, causing it to deviate from the typical trajectory exhibited by healthy individuals. The same complex mechanisms may explain the pathogenesis of cognitive impairment often seen in HIV, especially in motoric and executive domains. These new insights into the potential disruption of interactions between brain aging and glymphatic clearance in HIV patients shift our understanding of HIV pathology and could aid in developing new therapeutic targets for addressing HIV-related pathology, contrary to previous approaches.

## Data Availability

The raw data supporting the conclusions of this article will be made available by the authors, without undue reservation.
