# Supplementary material for: Human immunodeficiency virus accelerates brain aging and disrupts the trajectory of glymphatic clearance in aging brain
Source: Front Psychiatry. 2025 May 28;16:1509093. doi: 10.3389/fpsyt.2025.1509093 (PMC12152579; doi:10.3389/fpsyt.2025.1509093)
Supplement: Supplementary file 1 [file Table1.docx]

| **Supplementary Table S1** Patients’ Demographics and Clinical Characteristics | | | | | |
| --- | --- | --- | --- | --- | --- |
|  | **HC** | **HIV** | **HAND-Negative** | **HAND-positive(ANI)** | **Significance** |
| *N* | 45 | 100 | 59 | 41 |  |
| Age(years) | 34.47(6.75) | 31.74(6.90) | 32.63(7.59) | 30.27(5.90) | N/S |
| Range | 24-52 | 20-53 | 20-53 | 21-48 | — |
| Sex(M/F) | 41M, 4F | 96M, 4F | 56M, 3F | 40M,1F | — |
| Ethnicity (A/NA) | 45A | 100A |  |  |  |
| **ART status** |  |  |  |  |  |
| Current CD4 (cells/μL) | — | 493.62(196.13) | 510.95(183.53) | 487.89(210.66) | N/S |
| Range( CD4) | — | 8-1094 | 147-1094 | 65.77-923.57 | *—* |
| CD4/CD8 Ratio | — | 0.63(0.43) | 0.63(0.33) | 0.67(0.57) | N/S |
| Range | — | 0.03-2.33 | 0.24-1.55 | 0.13-2.33 | *—* |
| Viral Load (DET/TND) | — | 79TND (79%) | 48TND (80.70%) | 34TND (82.93%) | — |
| Duration of infection (years) | — | 0-10.64 | 0-10.64 | 0-7.46 | — |
| Current Treatment Regimen | — | TDF+3TC+EFV | TDF+3TC+EFV | TDF+3TC+EFV | — |
| Adherence to Treatment | — | AD (100%) | AD (100%) | AD (100%) | — |
| Duration on ART | — | 0-10.64 | 0-10.64 | 0-7.46 | — |
| Change in Treatment | — | NT | NT | NT | — |
| Side effects or Complication | — | NSE | NSE | NSE | — |

Values are displayed as mean (standard deviation) or range unless otherwise noted. M, male; F, female; A, Asian ; N/S, not significant at *P* = 0.05; DET, detectable viral load; TND, undetectable viral load, TDF, tenofovir; 3TC, lamivudine; EFV, efavirenz; NT, no change of treatment, NSE, no side effect detected,
